# Clinical characteristics and biomarker profile in early- and late-onset Alzheimer’s disease: the Shanghai Memory Study

**DOI:** 10.1093/braincomms/fcaf015

**Published:** 2025-01-15

**Authors:** Jie Wu, Jing Wang, Zhenxu Xiao, Jiaying Lu, Xiaoxi Ma, Xiaowen Zhou, Yuhan Wu, Xiaoniu Liang, Li Zheng, Ding Ding, Huiwei Zhang, Yihui Guan, Chuantao Zuo, Qianhua Zhao, the Shanghai Memory Study, Qianhua Zhao, Chuantao Zuo, Ding Ding, Yihui Guan, Huiwei Zhang, Jiaying Lu, Weiqi Bao, Li Zheng, Xiaoniu Liang, Jing Wang, Zhenxu Xiao, Xiaoxi Ma, Jie Wu, Jie Wang, Xiaowen Zhou

**Affiliations:** Institute and Department of Neurology, Huashan Hospital, Fudan University, Shanghai 200040, China; National Clinical Research Center for Aging and Medicine, Huashan Hospital, Fudan University, Shanghai 200040, China; National Center for Neurological Disorders, Huashan Hospital, Fudan University, Shanghai 200040, China; National Clinical Research Center for Aging and Medicine, Huashan Hospital, Fudan University, Shanghai 200040, China; Department of Nuclear Medicine and PET Center, Huashan Hospital, Fudan University, Shanghai 200235, China; Institute and Department of Neurology, Huashan Hospital, Fudan University, Shanghai 200040, China; National Clinical Research Center for Aging and Medicine, Huashan Hospital, Fudan University, Shanghai 200040, China; National Center for Neurological Disorders, Huashan Hospital, Fudan University, Shanghai 200040, China; National Clinical Research Center for Aging and Medicine, Huashan Hospital, Fudan University, Shanghai 200040, China; Department of Nuclear Medicine and PET Center, Huashan Hospital, Fudan University, Shanghai 200235, China; Institute and Department of Neurology, Huashan Hospital, Fudan University, Shanghai 200040, China; National Clinical Research Center for Aging and Medicine, Huashan Hospital, Fudan University, Shanghai 200040, China; National Center for Neurological Disorders, Huashan Hospital, Fudan University, Shanghai 200040, China; Institute and Department of Neurology, Huashan Hospital, Fudan University, Shanghai 200040, China; National Clinical Research Center for Aging and Medicine, Huashan Hospital, Fudan University, Shanghai 200040, China; National Center for Neurological Disorders, Huashan Hospital, Fudan University, Shanghai 200040, China; Institute and Department of Neurology, Huashan Hospital, Fudan University, Shanghai 200040, China; Institute and Department of Neurology, Huashan Hospital, Fudan University, Shanghai 200040, China; Institute and Department of Neurology, Huashan Hospital, Fudan University, Shanghai 200040, China; Institute and Department of Neurology, Huashan Hospital, Fudan University, Shanghai 200040, China; National Clinical Research Center for Aging and Medicine, Huashan Hospital, Fudan University, Shanghai 200040, China; National Center for Neurological Disorders, Huashan Hospital, Fudan University, Shanghai 200040, China; National Clinical Research Center for Aging and Medicine, Huashan Hospital, Fudan University, Shanghai 200040, China; Department of Nuclear Medicine and PET Center, Huashan Hospital, Fudan University, Shanghai 200235, China; National Clinical Research Center for Aging and Medicine, Huashan Hospital, Fudan University, Shanghai 200040, China; Department of Nuclear Medicine and PET Center, Huashan Hospital, Fudan University, Shanghai 200235, China; National Clinical Research Center for Aging and Medicine, Huashan Hospital, Fudan University, Shanghai 200040, China; Department of Nuclear Medicine and PET Center, Huashan Hospital, Fudan University, Shanghai 200235, China; Institute and Department of Neurology, Huashan Hospital, Fudan University, Shanghai 200040, China; National Clinical Research Center for Aging and Medicine, Huashan Hospital, Fudan University, Shanghai 200040, China; National Center for Neurological Disorders, Huashan Hospital, Fudan University, Shanghai 200040, China; MOE Frontiers Center for Brain Science, Fudan University, Shanghai 200030, China

**Keywords:** Alzheimer’s disease, plasma p-tau181, plasma NfL, PET, *APOE*

## Abstract

Early-onset Alzheimer’s disease constitutes ∼5–10% of Alzheimer’s disease. Its clinical characteristics and biomarker profiles are not well documented. To compare the characteristics covering clinical, neuropsychological and biomarker profiles between patients with early- and late-onset Alzheimer’s disease, we enrolled 203 patients (late-onset Alzheimer’s disease = 99; early-onset Alzheimer’s disease = 104) from a Chinese hospital-based cohort, the Shanghai Memory Study. A full panel of plasma biomarkers under the amyloid/tau/neurodegeneration framework including plasma amyloid beta 40, amyloid beta 42, total-tau, neurofilament light chain and phosphorylated tau 181 were assayed using ultra-sensitive Simoa technology. Seventy-five patients underwent an amyloid molecular positron emission tomography scan whereas 43 received comprehensive amyloid, Tau deposition and hypometabolism analysis. Clinical features, plasma and imaging biomarkers were compared cross-sectionally. Compared to those with late-onset Alzheimer’s disease, patients with early-onset Alzheimer’s disease presented more severe impairment in language function, lower frequency of *APOE* ɛ4 and lower levels of plasma neurofilament light chain (all *P* < 0.05). The plasma phosphorylated tau 181 concentration and phosphorylated tau 181/amyloid beta 42 ratios were higher in early-onset Alzheimer’s disease than in late-onset Alzheimer’s disease (all *P* < 0.05). More severe Tau deposition as indicated by ^18^F-florzolotau binding in the precuneus, posterior cingulate cortex and angular gyrus was observed in the early-onset Alzheimer’s disease group. Plasma phosphorylated tau 181 was associated with earlier age at onset and domain-specific cognitive impairment, especially in patients with early-onset Alzheimer’s disease. We concluded that patients with early-onset Alzheimer’s disease differed from late-onset Alzheimer’s disease in cognitive performance and biomarker profile. A higher burden of pathological tau was observed in early-onset Alzheimer’s disease and was associated with earlier age at onset and more profound cognitive impairment.

## Teaser

In this study, Wu *et al*. analysed plasma and PET imaging biomarkers related to amyloid, tau and neurodegenerative pathology in patients with early-onset and late-onset Alzheimer’s disease. The authors found that a greater burden of pathological tau was observed in individuals with early-onset Alzheimer’s disease.

## Graphical Abstract

**Graphical Abstract:**
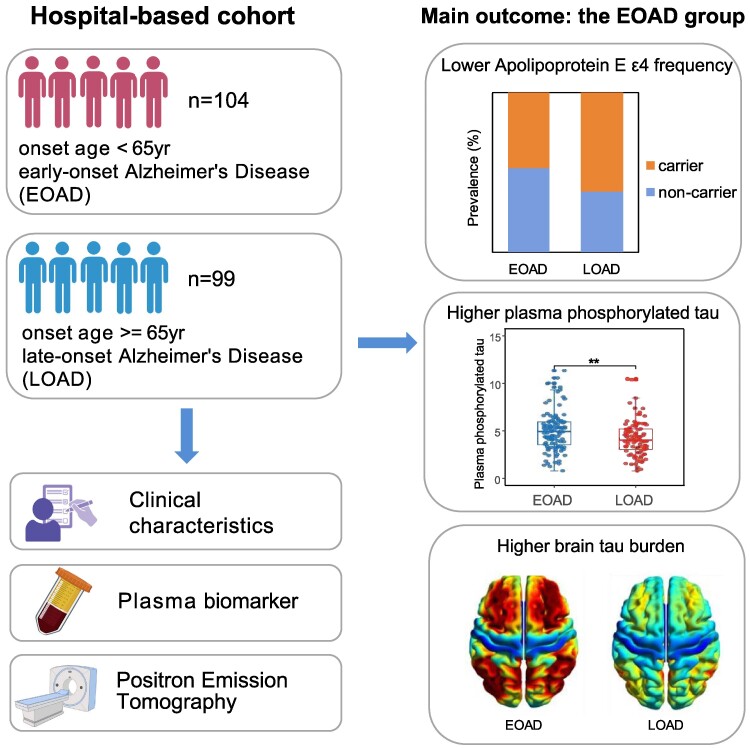

## Introduction

As the most common cause of dementia,^1^ Alzheimer’s disease is a progressive neurodegenerative disease that affects cognitive functions such as memory, language, executive and visuospatial function. Amyloid beta (Aβ) plaques and hyperphosphorylated tau (pTau) tangles are the main pathological hallmarks of Alzheimer's disease. Most patients with Alzheimer's disease develop symptoms after the age of 65, which had been defined as late-onset Alzheimer's disease (LOAD), meanwhile, ∼5–10% of the Alzheimer's disease patients develop symptoms before age 65, namely the early-onset Alzheimer's disease (EOAD).^2-4^

Compared to LOAD, patients with EOAD are more likely to have atypical clinical phenotypes. Approximately 25% of patients with EOAD present with focal cortical symptoms, such as apraxia, visual dysfunction, aphasia or executive dysfunction, while memory remains preserved at the initial stage.^4,5^ Furthermore, individuals with EOAD were reported to experience a more aggressive disease course than LOAD.^5^ Previous studies have reported that individuals with EOAD present different atrophy patterns,^6-8^ hypometabolism regions in ^18^FDG-positron emission tomography (PET),^9^ and CSF biomarker profiles^10^ compared with LOAD.

Fluid-based biomarkers, as well as molecular pathology in PET scans, have been established as promising measures for Alzheimer's disease^11,12^ and incorporated into the latest diagnostic criteria.^13,14^ The ATN (amyloid/tau/neurodegeneration) framework,^13^ established by the National Institute on Aging and the Alzheimer’s Association, had attracted attention in biomarker research. However, few prior studies have systematically evaluated the distinct biomarker profiles of EOAD in both peripheral blood and molecular images. Hereby, in a previously well-established hospital-based cohort, we explored the difference between EOAD and LOAD, covering clinical manifestation, plasma biomarkers and molecular pathology.

## Materials and methods

### Study participants and clinical diagnosis

The Shanghai Memory Study (SMS),^15,16^ established in 2009, was a hospital-based cohort study launched in the memory clinic of the Department of Neurology, Huashan Hospital, Fudan University, Shanghai, China. SMS was initially designed to investigate the risk factors, cognitive function, disease progression and neuroimaging of cognitive disorders with longitudinal follow-up.

In the current study, patients with Alzheimer's disease were consecutively recruited from SMS from 9 November 2018 to 28 January 2022. Dementia was diagnosed based on the Diagnostic and Statistical Manual of Mental Disorders, 4th edition (DSM-IV)^17^ and Alzheimer's disease according to the criteria from the National Institute of Neurological and Communicative Disorders and Stroke and the Alzheimer’s Disease and Related Disorders Association (NINCDS-ADRDA).^18^ Patients were excluded if they: (i) had cognitive impairment due to non-Alzheimer's disease aetiology including cerebral vascular disease, frontotemporal dementia, Lewy body dementia, Parkinson’s disease, etc.; (ii) had schizophrenia or mental retardation; (iii) could not cooperate with neuropsychological test; or (iv) refused the blood sample collection. All patients were divided into two groups according to the age at onset (AAO), the EOAD group (AAO < 65 years) and the LOAD group (AAO ≥ 65 years). A total of 203 of eligible patients (EOAD = 104, LOADO = 99) were recruited for the study.

This study was approved by the Medical Ethics Committee of Huashan Hospital, Fudan University, Shanghai, China. Written informed consent was obtained from all patients and their legal guardians.

### Demographic characteristics and clinical profile

Demographic characteristics including age, sex and years of education were acquired from all patients through detailed questionnaires. Comorbidities, including hypertension, diabetes and hyperlipidaemia, were based on self-report and medical records. The *Apolipoprotein E* (*APOE*) genotype was assessed by the TaqMan single nucleotide polymorphism method,^19^ and *APOE* ɛ4-positive was defined as having at least one ɛ4 allele.

Detailed clinical symptoms were collected by the neurologists at baseline. Patients were included in the frequency count of neuropsychiatric symptoms (NPS) if their medical history exhibited any of the following presentations: psychosis (delusion and hallucinations), agitation, aggression, depression, anxiety, apathy, disinhibition, motor disturbance, night-time behaviours, and appetite and eating problems.^20^

### Neuropsychological assessment

Each patient was administered a battery of comprehensive neuropsychological tests including: (i) Mini-Mental Status Exam (MMSE);^21^ (ii) Montreal Cognitive Assessment-Basic (MoCA-B);^22,23^ (iii) Auditory Verbal Learning test (AVLT);^24,25^ (iv) Trail-Making Test A (TMT-A) and B (TMT-B);^26^ (v) Rey–Osterrieth complex figure test (ROCFT);^15^ (vi) Boston Naming Test (BNT);^15,27^ (vii) Symbol Digit Modalities Test;^15,28^ (viii) Verbal Fluency Test;^25^ (ix) Clock Drawing Test;^15^ (x) Similarity Test;^29^ and (xi) Stroop colour-word test.^30^ All tests were conducted by professional psychometrists. The raw scores of each test were extracted to evaluate specific cognitive domains including memory, attention, visuospatial function, language and executive function (Supplementary Table 1).^31^

The severity of cognitive impairment was evaluated by the neurologists according to the Clinical Dementia Rating (CDR),^32,33^ which was a semi-structured inventory covering six cognitive, behavioural and functional aspects. The CDR-global score (CDR-GS) was calculated based on the Washington University CDR-assignment algorithm.

### Measurement of plasma biomarkers

Blood samples were collected from all patients at baseline in ethylenediamine tetraacetic acid plasma tubes and centrifuged at 1000 g, for 15 min (4°C). Plasma was aliquoted and stored at −80°C until the day of the test.

Plasma Aβ40, Aβ42, total-tau (t-tau), neurofilament light chain (NfL) and p-tau181 were measured using an ultra-sensitive Simoa technology (Quanterix, MA, USA) on the automated Simoa HD-X platform (GBIO, Hangzhou, China). The multiplex Neurology 3-Plex A, p-tau181 V2 and NfL assay kits were purchased from Quanterix and performed according to the manufacturer’s instructions for use. All patients’ disease status and clinical information were blind to all operators.

### Image acquisition and processing

Of the initial cohort, 75 subjects had a positive amyloid PET scan based on expert visual assessment. Among these, 57 patients underwent ^18^F-florzolotau PET (tau PET) and ^18^F-fluorodeoxyglucose (^18^F-FDG) PET. The PET scan was conducted using ligands ^18^F-florbetapir (^18^F-AV-45; 50–70 min post-injection) for amyloid, ^18^F-florzolotau (90–110 min post-injection) for tau and ^18^F-FDG (60–70 min post-injection) for hypometabolism. Static images were acquired on a Biograph mCT Flow PET/CT system (Siemens, Erlangen, Germany). T_1_-weighted MRI images were obtained with a 3.0-T horizontal magnet scanner (Discovery MR750; GE Medical Systems, Milwaukee, WI, USA) and were used for spatial normalization.

Raw Aβ PET images were visually interpreted using dedicated software (Siemens syngo. via) by two independent neuroradiologists (C.Z., more than 20 years of experience; J.L., more than 5 years of experience) blinded to clinical and laboratory data. Each participant was classified as either Aβ-positive (Aβ+) or Aβ-negative (Aβ−) according to the criteria proposed previously.^34^ A third expert (H.Z., more than 10 years of experience) was invited to review images in case of discrepancies; the final classification was based on majority voting.

Individual PET images were thoroughly coregistered to the T_1_-weighted MRI images, corrected for partial volume effects, and then normalized into the Montreal Neurological Institute standard space using the transformation matrix for each segmented T_1_-weighted MRI image. Tracer-specific reference regions were selected as whole cerebellum for ^18^F-florbetapir PET, cerebellar grey matter for ^18^F-florzolotau PET and pons for ^18^F-FDG PET. Standardized uptake value ratio (SUVR) images were obtained after reference region-based intensity normalization. The details of image acquisition and processing could be found in previous publications.^35,36^ Neuroimaging Images were processed via Statistical Parametric Mapping version 12 (SPM12; http://www.fil.ion.ucl.ac.uk/spm/software/spm12/) software in MATLAB (version 2018a, MathWorks, Natick, MA).

### Statistical analyses

Shapiro–Wilk test was conducted to assess the normality of the data distribution. Continuous data were summarized and shown as ‘mean [standard deviations (SDs)]’ for normally distributed data, and ‘median [interquartile range (IQR)]’ for non-normally distributed data. Categorical data were described as ‘frequency (percentage)’.

To compare the demographic and comorbid characteristics between the two groups, we utilized the Chi-square analysis or Fisher’s exact test for categorical data. For continuous data intergroup comparisons, we employed the *t*-test when the data met normal distribution assumptions, and the Mann–Whitney U-test was used when the data did not meet such assumptions. The comparison of plasma biomarkers was performed using analysis of covariance (ANCOVA), with the adjustment for gender, education, *APOE* genotype and CDR. Subgroup analysis was conducted to investigate whether the concentration of plasma biomarkers varied between EOAD and LOAD across different disease severity (mild, moderated, severe) as evaluated by CDR (CDR ≤ 1, CDR = 2, CDR = 3). Two linear regression models were conducted to investigate the relationship between AAO and plasma biomarker levels. Model 1 was a univariate linear regression model, while Model 2 adjusted for CDR, gender, education and *APOE* genotype. Partial correlation coefficients adjusted for sex, education, *APOE* genotype and CDR were calculated to determine the association between neuropsychological measurements and plasma biomarker concentrations.

To rule out the confounding effect of disease severity, the comprehensive image analysis between EOAD and LOAD was conducted only in the CDR = 1 subgroup. Mean images for ATN PET were generated separately for the EOAD and LOAD groups within the CDR = 1 subgroup (EOAD *n* = 23, LOAD *n* = 20) for visualization. Given that age was the primary factor investigated in the current study and cognition was controlled, the voxel-wise group comparisons only adjusted for sex and were conducted using SPM 12 with a two-sample *t*-test. The primary statistical threshold was set at *P* < 0.001, with a minimum cluster size of 10 voxels (uncorrected for multiple comparisons). Significance was set at *P* < 0.05. Data analysis was conducted using IBM SPSS statistics version 26 statistical software and programming language R (version 4.2.1). The heatmap was visualized by GraphPad Prism version 9.4.1 (681).

## Results

### Participants’ characteristics

The baseline characteristics of all 203 Alzheimer's disease patients enrolled in SMS are summarized in Table 1. The median AAO was 55.00 (IQR: 52.00–61.00) for the EOAD group and 71.00 (IQR: 68.00–77.00) for the LOAD group. Compared to the LOAD group, the EOAD group had a lower frequency of *APOE* ɛ4 (61.6% versus 47.1%, *P* = 0.038), lower education, higher prevalence of NPS, greater disease severity and pronounced impairments in global cognition and language (all *P* < 0.05). Seven patients in the EOAD group were diagnosed with atypical Alzheimer's disease (four patients with posterior cortical atrophy, one with logopenic primary progressive aphasia, one with frontal variant of Alzheimer's disease and one with Alzheimer's disease combined with corticobasal syndrome), one patient was diagnosed with atypical Alzheimer's disease (logopenic primary progressive aphasia) in the LOAD group, no significant difference was found on the proportion of atypical Alzheimer's disease (*P* = 0.083). No significant difference was found in gender and history of hypertension, diabetes and hyperlipidaemia (based on self-report and medical records).

**Table 1:** Baseline characters of participants

	EOAD (*n* = 104)	LOAD (*n* = 99)	*P*-value
Age at onset, median (IQR)	55.00 (52.00, 61.00)	71.00 (68.00, 77.00)	
Sex, female (%)	61 (58.7%)	55 (55.6%)	0.673
Years of education, median (IQR)	9.00 (5.00, 12.00)	9.00 (6.00, 12.00)	0.044
*APOE*4 carriers, *n* (%)	49 (47.1%)	61 (61.6%)	0.038
Clinical presentation			
Atypical Alzheimer's disease, *n* (%)	7 (6.7%)	1 (1.0%)	0.083^a^
Neuropsychiatric symptoms, *n* (%)	21 (20.3%)	9 (9.1%)	0.026
Neuropsychological tests			
MMSE score, median (IQR)	16.00 (9.25, 21.00)	19.00 (16.00, 22.00)	0.002
Z-memory	−0.45 (−0.66, 0.59)	−0.65 (−0.66, 0.59)	0.096
Z-attention	−0.53 (−0.72, 0.47)	−0.63 (−0.72, 1.07)	<0.001
Z-visuospatial function	−0.79 (−0.96, 0.73)	0.34 (−0.96, 1.34)	0.086
Z-language	0.02 (−1.00, 0.53)	0.53 (0.02, 1.04)	0.001
Z-executive function	−0.61 (−0.60, 0.55)	−0.61 (−0.61, 0.08)	0.641
CDR-GS			0.009
≤1	42 (40.4%)	56 (56.6%)	
2	34 (32.7%)	32 (32.3%)	
3	28 (26.9%)	11 (11.1%)	
Comorbidities			
Hypertension	29 (27.9%)	38 (38.4%)	0.112
Diabetes	10 (9.6%)	15 (15.2%)	0.287
Hyperlipidaemia	23 (22.1%)	30 (30.3%)	0.121
Family history of dementia	21 (21.0%)	24 (25.5%)	0.455

EOAD, early-onset Alzheimer’s disease; LOAD, late-onset Alzheimer’s disease; *APOE*, apolipoprotein E; MMSE, Mini-Mental State Examination; CDR-GS, Clinical Dementia Rating-global score; IQR, interquartile range.

^a^ *P*-value was calculated from Fisher’s exact test.

### Plasma biomarkers in EOAD and LOAD groups

**Figure 1:**
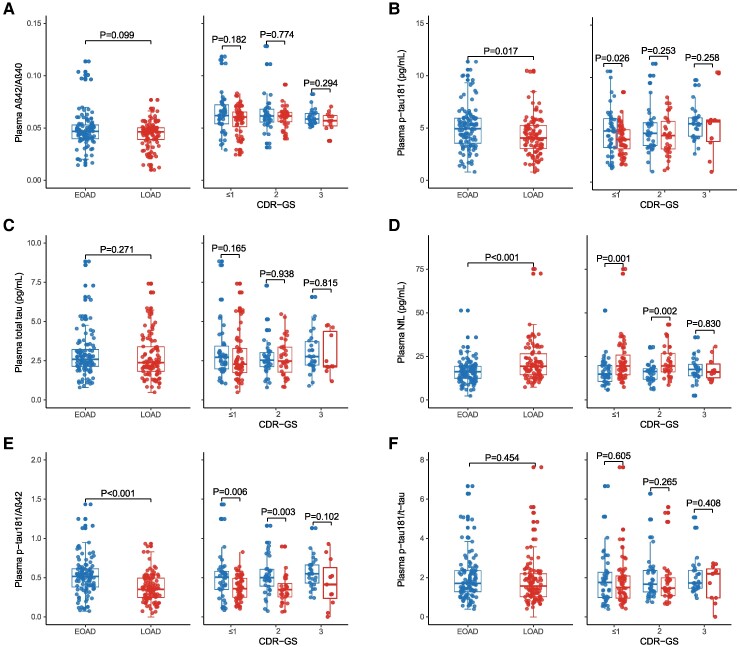
**Concentrations of plasma biomarkers among patients with different AAO.** (**A–F**) Comparison of plasma biomarkers in the total sample and CDR-based subgroups between EOAD and LOAD groups under the ATN framework. AAO, age at onset; CDR-GS, Clinical Dementia Rating-global score; Aβ, amyloid-beta protein; NfL, neurofilament light chain; P-tau181, tau phosphorylated at threonine 181. *P*-values were calculated based on log-transformed data. ANCOVA analysis was conducted for the group comparison. Sex, education, *APOE* genotype and CDR-GS were adjusted in the first analysis; sex, education and *APOE* genotype were adjusted in the subgroup analysis. Statistic values: F = 2.772, *P* = 0.099; F_(CDR≤1)_ = 1.745, *P* = 0.182; F_(CDR = 2)_ = 0.083, *P* = 0.774; F_(CDR = 3)_ = 1.155, *P* = 0.294, respectively, for **A**. F = 5.856, *P* = 0.017; F_(CDR≤1)_ = 7.432, *P* = 0.026; F_(CDR = 2)_ = 1.700, *P* = 0.253; F_(CDR = 3)_ = 0.050, *P* = 0.258 for **B**. F = 0.843, *P* = 0.271; F_(CDR≤1)_ = 1.867, *P* = 0.165; F_(CDR = 2)_ = 0.054, *P* = 0.938; F_(CDR = 3)_ = 0.032, *P* = 0.815, respectively, for **C**. F = 16.302, *P* < 0.001; F_(CDR≤1)_ = 10.093, *P* = 0.001; F_(CDR = 2)_ = 10.285, *P* = 0.002; F_(CDR = 3)_ = 0.004, *P* = 0.830, respectively, for **D**. F = 20.709, *P* < 0.001; F_(CDR≤1)_ = 11.217, *P* = 0.006; F_(CDR = 2)_ = 12.989, *P* = 0.003; F_(CDR = 3)_ = 0.935, *P* = 0.102, respectively, for **E**. F = 0.887, *P* = 0.454; F_(CDR≤1)_ = 0.570, *P* = 0.605; F_(CDR = 2)_ = 0.741, *P* = 0.265; F_(CDR = 3)_ = 0.364, *P* = 0.408, respectively, for **F**.

As shown in Fig. 1 and Supplementary Table 2, compared to the LOAD group, the EOAD group presented with higher levels of plasma p-tau181 (*P* = 0.017) (Fig. 1B) and p-tau181/Aβ42 ratio (*P* < 0.001) (Fig. 1E), and lower level of NfL (*P* < 0.001) (Fig. 1D). Regarding the bias of disease severity at baseline, subgroup analyses based on CDR score were conducted to investigate the profile of plasma biomarkers in EOAD and LOAD patients with different disease severity. The EOAD group showed a higher p-tau181 concentration in the CDR ≤ 1 subgroup, a lower level of plasma NfL in the CDR ≤ 1 and CDR = 2 subgroups, and higher p-tau181/Aβ42 in the CDR ≤ 1 and CDR = 2 subgroups (all *P* < 0.05). No difference was found in plasma Aβ42/Aβ40 (Fig. 1A), t-tau (Fig. 1C) and p-tau181/t-tau (Fig. 1F).

### PET imaging biomarkers

**Figure 2:**
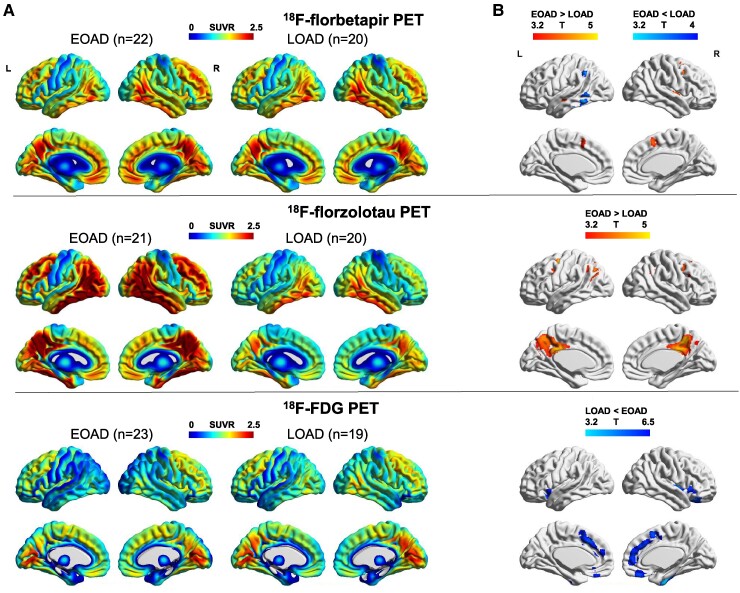
**Mean images and group comparison of PET imaging biomarker.** (**A**) Mean interpolated surface projections of average SUVR maps of EOAD and LOAD groups for amyloid (^18^F-florbetapir, upper), tau (^18^F-florzolotau, middle) and hypometabolism (^18^F-FDG, lower) PET. (**B**) Voxel-wise comparisons were performed using a two-sample *t*-test, adjusting for sex. Significant maps were identified at a threshold of *P* < 0.0001 with a minimum cluster size of 10 voxels, uncorrected for multiple comparisons. EOAD, early-onset Alzheimer’s disease; LOAD, late-onset Alzheimer’s disease; SUVR, standardized uptake value ratio; PET, positron emission tomography.

To avoid the potential bias by disease severity, the comparison for PET images was restricted to the CDR = 1 subgroup. The gender, education and MMSE score were not significantly different between the two subgroups (All *P* > 0.05) (Supplementary Table 3). Voxel-wise comparisons covering Amyloid, Tau and Neurodegeneration PET markers were conducted. Mean images for each modality were displayed (Fig. 2). There was no significant difference between EOAD and LOAD in the global amyloid deposition (Supplementary Fig. 1A), but the ^18^F-florbetapir binding was slightly higher in the right superior frontal and temporal cortical in EOAD (Supplementary Table 4), whereas the SUVRs were modestly higher in the left medial and inferior temporal gyrus in LOAD (Fig. 2B, upper; Supplementary Table 5). For ^18^F-florzolotau PET, the mean image showed a more pronounced and widespread Tau deposition in EOAD across the entire cerebral cortex in both hemispheres, only sparing the central sulcus (Fig. 2A, middle). Notably, in Braak III and IV and Braak V and VI regions, the EOAD group had greater ^18^F-florzolotau binding than LOAD (Supplementary Fig. 1B and C). Group-level comparison using SPM revealed that the most significantly discrepant areas lied in the precuneus, posterior cingulate cortex and angular gyrus (Fig. 2B, middle; Supplementary Table 6). For FDG-PET, compared to the EOAD group, the LOAD group showed greater hypometabolism in the right superior frontal gyrus, and right lateral occipitotemporal region (Figure 2B, lower; Supplementary Table 7).

### Plasma biomarkers and AAO

Regression analysis between AAO and plasma biomarkers was conducted (Table 2). In the univariate model, higher NfL was significantly associated with older AAO (*B* = 0.007, 95% CI 0.004–0.010, standardized *B* = 0.343, *P* < 0.001), while higher plasma p-tau181 concentration (*B* = −0.004, 95% CI −0.007 to −0.001, standardized *B* = −0.205, *P* = 0.004) and p-tau181/Aβ42 (*B* = −0.008, 95% CI −0.012 to −0.005, standardized *B* = −0.331, *P* < 0.001) were associated with younger AAO. After adjusting for CDR, gender, education and *APOE* genotype, these significant associations remained. In the subgroup analysis within the EOAD group, the univariate analysis revealed a significant negative association between p-tau181/Aβ42 and AAO (*B* = −0.010, 95% CI −0.019 to −0.001, standardized *B* = −0.219, *P* = 0.025), and a marginal association between plasma p-tau181 (*B* = −0.007, 95% CI −0.015–0, standardized *B* = −0.190, *P* = 0.054) and AAO. In the LOAD group, plasma NfL was positively associated with AAO (*B* = 0.008, 95% CI 0.001–0.016, standardized *B* = 0.224, *P* = 0.027). However, no significant association for subgroup analysis was observed in the multiple regression model.

**Table 2:** Multiple linear regression of plasma biomarkers and AAO

Plasma biomarkers	Model 1^a^			Model 2^b^		
	*B* (95% CI)	Standardized *B*	*P*-value	*B* (95% CI)	Standardized *B*	*P*-value
Total						
NfL	0.007 (0.004, 0.010)	0.343	<0.001	0.007 (0.004, 0.010)	0.339	<0.001
P-tau181	−0.004 (−0.007, −0.001)	−0.205	0.004	−0.004 (−0.007, −0.001)	−0.182	0.006
P-tau181/Ab42	−0.008 (−0.012, −0.005)	−0.331	<0.001	−0.008 (−0.012, −0.005)	−0.325	<0.001
EOAD						
NfL	−0.003 (−0.010, 0.004)	−0.082	0.406	−0.003 (−0.011, 0.005)	−0.075	0.475
P-tau181	−0.007 (−0.015, 0.000)	−0.190	0.054	−0.005 (−0.013, 0.003)	−0.127	0.226
P-tau181/Ab42	−0.010 (−0.019, −0.001)	−0.219	0.025	−0.009 (−0.019, 0.001)	−0.195	0.064
LOAD						
NfL	0.008 (0.001, 0.016)	0.224	0.027	0.006 (−0.002, 0.014)	0.162	0.145
P-tau181	0.000 (−0.008, 0.007)	−0.003	0.977	0.000 (−0.008, 0.008)	0.007	0.948
P-tau181/Ab42	−0.005 (−0.014, 0.003)	−0.128	0.213	−0.005 (−0.014, 0.004)	−0.117	0.276

AAO, age at onset; EOAD, early-onset Alzheimer’s disease; LOAD, late-onset Alzheimer’s disease; Aβ, amyloid-beta protein; NfL, neurofilament light chain; P-tau181, tau phosphorylated at threonine 181.

^a^Model 1 was a univariate linear regression model.

^b^In Model 2, data were adjusted for CDR-global score, sex, education duration and *APOE* genotype. The concentration of plasma biomarkers was log-transformed.

### Plasma biomarkers and domain-specific cognition

**Figure 3:**
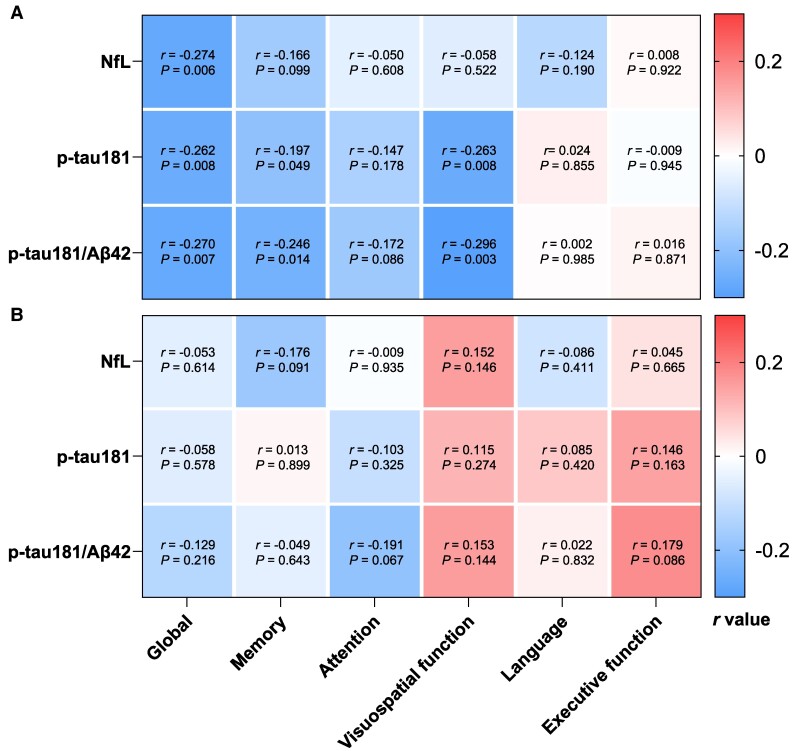
**Correlations between plasma biomarker concentrations and cognitive domains.** (**A**) The correlations between plasma biomarker concentrations and cognitive domains in the EOAD group; (**B**) the correlations between plasma biomarker concentrations and cognitive domains in the LOAD group. The correlation coefficients (*r*) and *P*-values were obtained from partial correlation analysis, adjusting for sex, CDR-GS, education duration and *APOE* genotype. Plasma biomarker concentrations were log-transformed before analysis. Aβ, amyloid-beta protein; NfL, neurofilament protein light chain; p-tau181, tau phosphorylated at threonine 181.

As shown before, plasma biomarkers including p-tau181, NfL and p-tau181/Aβ42 ratio, differed significantly between EOAD and LOAD groups. We then performed further correlation analysis between six domain-specific cognitive performances and the three plasma biomarkers. As shown in Fig. 3, in patients with EOAD, all three plasma biomarkers were negatively associated with cognitive function. Specifically, NfL was negatively correlated with global cognition (*r* = −0.274, *P* = 0.006) and p-tau181 was inversely associated with global cognition (*r* = −0.262, *P* = 0.008), memory (*r* = −0.197, *P* = 0.049) and visuospatial function (*r* = −0.263, *P* = 0.008), while p-tau181/Aβ42 was negatively linked to global cognition (*r* = −0.270, *P* = 0.007), memory (*r* = −0.246, *P* = 0.014) and visuospatial function (*r* = −0.296, *P* = 0.003) (Fig. 3A). However, no significant correlation between plasma biomarkers and any cognitive domain was observed in the LOAD group (Fig. 3B).

## Discussion

In the current study, clinical characteristics, as well as full panel biomarker profiles were extensively explored among patients with EOAD and LOAD, which yielded three main findings. First, EOAD patients exhibited more severe language impairment, more frequent NPS and lower frequency of *APOE* ɛ4 allele. Second, patients with EOAD showed increased plasma p-tau181, p-tau181/Aβ42 ratio and reduced plasma NfL. Multi-modal PET analysis also indicated a higher tau burden but less neurodegeneration in EOAD. Lastly, pTau-related plasma biomarkers were associated with earlier AAO and worse cognitive function in EOAD but not in LOAD.

As the most important genetic risk factor for sporadic AD, the *APOE* ɛ4 allele was known to be associated with an increased lifetime risk of developing Alzheimer's disease.^37^*APOE* ɛ4 especially *APOE* ɛ4 homozygosity was reported to be associated with greater Alzheimer's disease pathology.^38-40^ In the current cohort, the frequency of *APOE* ɛ4 in EOAD was significantly lower than in LOAD, which was in line with previous research.^41,42^ However, despite the lower frequency of *APOE* ɛ4, the EOAD group exhibited a greater tau burden, which indicated that other potential genetic^43^ or environmental^44,45^ factors other than *APOE* ɛ4 might contribute to the pathogenesis of EOAD.

Consistent with prior studies,^3,4,46,47^ patients in the EOAD group exhibited poorer language function in the current study, which might be attributed to the higher proportion of atypical Alzheimer's disease cases within the EOAD group. According to previous studies, approximately one-third of EOAD patients presented with atypical syndromes, which was much higher than in LOAD.^4,48,49^ However, in the current study, the frequency of atypical Alzheimer's disease was relatively lower than in the previous studies. One possible reason was that the more advanced cognitive impairment of EOAD in the current study might mask the initial clinical features that were crucial to atypical Alzheimer's disease diagnosis. Additionally, The EOAD group showed a higher frequency of NPS at baseline, aligning with previous studies.^50-52^

Full panel biomarker analysis covering plasma measure and molecular image were conducted across the ATN scheme. In the current study, the EOAD group exhibited elevated plasma p-tau181 levels and p-tau181/Aβ42 ratio, which was consistent with previous findings. Lv *et al*.^53^ measured plasma biomarkers in 106 patients with EOAD and 88 patients with LOAD and found that the EOAD group had higher plasma p-tau181 concentrations, while no difference was found in t-tau, Aβ42/Aβ40 and NfL. Since the plasma p-tau181 and p-tau181/Aβ42 ratio were significantly associated with amyloid pathology,^54,55^ tau pathology^56,57^ and brain atrophy,^55^ our results supported a higher Alzheimer's disease pathology burden in EOAD. Interestingly, the PET analysis revealed no difference in the global amyloid deposition, less severe neurodegeneration in EOAD, but a significantly higher Tau deposition in EOAD, which was in accordance with previous findings.^58-60^ Xu *et al*.^58^ and Cho *et al*.^60^ reported that amyloid deposition did not significantly differ between the EOAD and LOAD groups. Tanner *et al*.^59^ reported higher tau PET SUVR in the EOAD group. Meanwhile, we noticed a higher plasma NfL concentration in the LOAD group. As plasma NfL was a sensitive indicator for neuron death and axon damage,^61^ the finding suggested more severe neurodegeneration in LOAD. At the same time, plasma NfL was reported to be correlated with various confounding factors such as age and comorbidities,^62-64^ which could also explain the higher NfL concentration in the LOAD group. Together with the findings of plasma and PET imaging biomarkers, we hypothesized that the EOAD patients had higher tau burden and patients with LOAD had greater neurodegeneration.

In further analysis, we investigated the associations between AAO and the three plasma biomarkers that exhibited distinct concentrations between the EOAD and LOAD groups. Across the entire cohort, all three biomarkers were significantly associated with AAO, consistent with the outcomes observed in the group comparison. In the EOAD group, p-tau181 and the p-tau181/Aβ42 ratios were negatively associated with AAO, with no significant association observed between NfL and AAO. Conversely, only plasma NfL was associated with AAO in the LOAD group. These findings, combined with the results from ATN biomarker group comparisons, suggested that tau pathology, rather than neurodegeneration, plays a crucial role in EOAD.

Partial correlation analysis showed higher plasma p-tau181 was associated with worse cognitive function, especially in the EOAD group, which suggested increased tau pathology might contribute to a more aggressive disease course. However, no significant association was observed in the LOAD group. Results were mixed when examining the correlation between plasma biomarkers and cognitive domains.^31,65^ The paradoxical results may be attributed to the diverse distribution in clinical and pathological conditions.

The findings should be interpreted within the context of several limitations. First, given the more rapidly progressive disease course, the EOAD patients often suffered a more severe cognitive dysfunction at the first clinical visit. The unbalanced disease severity might potentially introduce bias into the results. To mitigate this, subgroup analysis stratified by CDR, as well as the statistical adjustment for disease severity including multiple regression model, partial correlation and ANCOVA analysis were conducted. Second, in the current study, the percentage of atypical Alzheimer's disease was lower than previously reported limiting the representative of the results. Moreover, only the *APOE* genotype was investigated in the current study, other AD-related genes such as PSENs/APP, sortilin-related receptor 1 and triggering receptor expressed on myeloid cells 2 were not analysed. Additionally, most participants were diagnosed clinically, and only part of them received amyloid and tau PET confirmation, and the majority of the patients who underwent PET were in the early stage of Alzheimer's disease. The limited sample size precluded the investigation of PET imaging biomarkers in patients in the late stage of Alzheimer's disease, as well as the relationship between PET imaging and plasma biomarkers, and the impact of PET imaging biomarkers on the association between plasma biomarkers and cognition. Future studies expanding samples with pathology-confirmed patients are warranted. Furthermore, the data of AAO was collected from the questionnaire and the recall bias might influence the classification of EOAD and LOAD. However, AAO in our study was collected through detailed interviews with patients and their caregivers and was not only served as a binary variable (for EOAD versus LOAD classification) but also as a continuous variable in our regression analyses, which helped to alleviate the potential distortions caused by recall bias. Lastly, in the latest criteria^14^ for diagnosis and staging of Alzheimer's disease, the biomarker framework was updated to the ATX system (amyloid, tau, neurodegeneration, inflammation, vascular and α-synuclein), however, only amyloid, tau and neurodegeneration were discussed in this study. Further investigation on the biomarkers of inflammation, vascular and α-synuclein will be conducted.

## Conclusion

The present study illustrated distinctions between patients with EOAD and LOAD in terms of cognitive performance, plasma biomarkers and molecular pathology. A higher Tau burden might be the most remarkable feature of EOAD and contribute to its aggressive clinical course. To further elucidate the underlying mechanisms contributing to the heterogeneity of Alzheimer's disease, future investigations should explore genetic variations in EOAD and LOAD.

## Supplementary Material

fcaf015_Supplementary_Data

## Data Availability

Fully anonymized data will be shared upon request from qualified investigators, subject to approval by the China Human Genetic Resources Administration Office. Data transfer will have to comply with the Regulations of the People’s Republic of China.

## Appendix

The Shanghai Memory Study consortium: Qianhua Zhao, Chuantao Zuo, Ding Ding, Yihui Guan, Huiwei Zhang, Jiaying Lu, Weiqi Bao, Li Zheng, Xiaoniu Liang, Jing Wang, Zhenxu Xiao, Xiaoxi Ma, Jie Wu, Jie Wang, Xiaowen Zhou.
