# Supplementary material for: Clinical characteristics and biomarker profile in early- and late-onset Alzheimer’s disease: the Shanghai Memory Study
Source: Brain Commun. 2025 Jan 15;7(1):fcaf015. doi: 10.1093/braincomms/fcaf015 (PMC11756380; doi:10.1093/braincomms/fcaf015)
Supplement: fcaf015_Supplementary_Data [file fcaf015_supplementary_data.docx]

**Supplementary Table 1. Domain-specific cognition extracted from neuropsychological tests in the** current study

| **Global cognition** | **Memory** | **Attention** | **Visuospatial function** | **Language** | **Executive function** |
| --- | --- | --- | --- | --- | --- |
| MMSE | 'delayed recall'  in MMSE | 'registration'  in MMSE | 'overlapping imaging'  in MMSE | 'naming'  in MMSE | 'trail making' & 'similarity'  in MoCA-B |
|  | 'delayed recall'  in MoCA-B | 'attention'  in MoCA-B | 'copy'  in Rey-Osterrieth Complex Figure | 'naming'  in MoCA-B | 'part B completion time'*  in Trail Making Test (TMT) |
|  | 'delayed recall'  in Auditory Verbal Learning Test | Symbol Digit Modalities Test | Clock drawing test | Boston Naming Test | Stroop color-word test |
|  |  | 'part A completion time'*  in Trail Making Test (TMT) |  | Verbal fluency test |  |

Note: The score of each test was transformed into percentage of correct answer (%) in all participants [1]

*The performance time values of TMT were divided into three categories to calculate the accuracy according to previously published normative data [2]. The TMT time more than mean + 1.5 standard deviation (SD) was regarded as poor executive function and was assigned 0 score. The TMT time less than mean – 1.5 SD was regarded as good executive function and was assigned 2 scores. Other participants were assigned 1 score. Then, the new TMT scores were also converted to accuracy and *Z*-scores.

Abbreviations: MMSE, mini-mental state examination; MoCA-B, Montreal Cognitive Assessment-Basic; TMT, Trail Making Test.

[1] Nathan PJ, Lim YY, Abbott R, Galluzzi S, Marizzoni M, Babiloni C, Albani D, Bartres-Faz D, Didic M, Farotti L, Parnetti L, Salvadori N, Müller BW, Forloni G, Girtler N, Hensch T, Jovicich J, Leeuwis A, Marra C, Molinuevo JL, Nobili F, Pariente J, Payoux P, Ranjeva JP, Rolandi E, Rossini PM, Schönknecht P, Soricelli A, Tsolaki M, Visser PJ, Wiltfang J, Richardson JC, Bordet R, Blin O, Frisoni GB (2017) Association between CSF biomarkers, hippocampal volume and cognitive function in patients with amnestic mild cognitive impairment (MCI). *Neurobiol Aging* **53**, 1-10.

[2] Ding D, Zhao Q, Guo Q, Meng H, Wang B, Luo J, Mortimer JA, Borenstein AR, Hong Z Prevalence of mild cognitive impairment in an urban community in China: a cross-sectional analysis of the Shanghai Aging Study.*Alzheimers Dement. Mar* 2015;11(3):300-9.e2. doi:10.1016/j.jalz.2013.11.002

**Supplementary Table 2: the estimates of plasma biomarkers**

|  | **EOAD** | **LOAD** | **P value** |
| --- | --- | --- | --- |
| Aβ42/Aβ40 | | | |
| Total sample | 0.06 (0.00) | 0.05 (0.00) | 0.099 |
| CDR=<1 | 0.06 (0.00) | 0.06 (0.00) | 0.182 |
| CDR=2 | 0.06 (0.00) | 0.06 (0.00) | 0.774 |
| CDR=3 | 0.06 (0.00) | 0.05 (0.00) | 0.294 |
| p-tau 181 (pg/ml) | | | |
| Total sample | 4.95 (0.20) | 4.14 (0.18) | 0.017 |
| CDR=<1 | 4.67 (0.34) | 3.95 (0.19) | 0.026 |
| CDR=2 | 4.86 (0.39) | 4.25 (0.33) | 0.253 |
| CDR=3 | 5.48 (0.30) | 4.82 (0.93) | 0.258 |
| Total-tau (pg/ml) | | | |
| Total sample | 2.93 (0.14) | 2.75 (0.15) | 0.271 |
| CDR=<1 | 3.06 (0.27) | 2.81 (0.21) | 0.165 |
| CDR=2 | 2.68 (0.21) | 2.69 (0.22) | 0.938 |
| CDR=3 | 3.03 (0.24) | 2.53 (0.37) | 0.815 |
| NfL (pg/ml) | | | |
| Total sample | 16.48 (0.67) | 21.95 (1.14) | <0.001 |
| CDR=<1 | 16.28 (1.22) | 22.64 (1.73) | 0.001 |
| CDR=2 | 15.67 (0.94) | 21.75 (1.57) | 0.002 |
| CDR=3 | 17.78 (1.28) | 18.48 (2.29) | 0.830 |
| p-tau181/Aβ42 | | | |
| Total sample | 0.52 (0.03) | 0.37 (0.02) | <0.001 |
| CDR=<1 | 0.49 (0.05) | 0.37 (0.02) | 0.006 |
| CDR=2 | 0.52 (0.04) | 0.35 (0.03) | 0.003 |
| CDR=3 | 0.55 (0.04) | 0.44 (0.09) | 0.102 |
| p-tau181/t-tau | | | |
| Total sample | 1.97 (0.11) | 1.82 (0.12) | 0.454 |
| CDR=<1 | 1.88 (0.20) | 1.80 (0.16) | 0.605 |
| CDR=2 | 2.03 (0.21) | 1.84 (0.22) | 0.265 |
| CDR=3 | 2.05 (0.17) | 1.88 (0.27) | 0.408 |

Note: Values are expressed as mean (SD), and P value were calculated using ANCOVA. Sex, education, APOE genotype, and CDR-GS were adjusted in the total-sample analysis; sex, education, and APOE genotype were adjusted in the subgroup analysis.

**Supplementary Table 3. Baseline characteristic of patient enrolled in the image analysis**

|  | **EOAD (n=23)** | **LOAD (n=20)** | **P value** |
| --- | --- | --- | --- |
| Age at onset, median (IQR) | 56.50 (52.00, 61.50) | 70.00 (67.00, 74.75) |  |
| Gender, female (%) | 9(39.1%) | 12(60.0%) | 0.137 |
| Years of education, median (IQR) | 9.50 (8.00, 12.75) | 12.00 (8.25, 13.75) | 0.196 |
| APOE4 carriers, n (%) | 9(39.1%) | 14(70.0%) | 0.032 |
| MMSE score, median (IQR) | 21.00(20.00, 22.75) | 21.00 (19.00, 22.00) | 0.294 |

Abbreviations: EOAD: early-onset Alzheimer’s disease, LOAD: late-onset Alzheimer’s disease, APOE: apolipoprotein E, MMSE: Mini-Mental State Examination, IQR: interquartile range.

**Supplementary Table 4. Volume summary of voxel-wise imagine analysis (^18^F-florbetapir PET; EOAD >LOAD)**

| **Label** | **Cluster** | **Nb Vx Cluster** | **Label** | **Nb Vx Label** |
| --- | --- | --- | --- | --- |
| Frontal_Sup_2_R | 55.81 | 43 | 0.47 | 5126 |
| Frontal_Mid_2_R | 44. 19 | 43 | 0.39 | 4860 |
| Insula_R | 74.14 | 58 | 2.43 | 1770 |
| Frontal_Mid_2_R | 36.36 | 11 | 0.08 | 4860 |
| Temporal_Sup_L | 100.00 | 29 | 1. 26 | 2296 |
| Supp_Motor_Area_R | 91.43 | 35 | 1. 35 | 2371 |
| Supp_Motor_Area | 5. 71 | 35 | 0.09 | 2147 |
| Cingulate_Mid_R | 2.86 | 35 | 0.05 | 2203 |
| Fusiform_R | 91.67 | 12 | 0.44 | 2518 |
| Temporal_ Inf_R | 8.33 | 12 | 0.03 | 3557 |
| Supp_Motor_Area_L | 90.48 | 21 | 0.88 | 2147 |
| Cingulate_Mid_L | 9.52 | 21 | 0.10 | 1941 |

**Supplementary Table 5. Volume summary of voxel-wise imagine analysis (^18^F-florbetapir PET; LOAD >EOAD)**

| **Label** | **%Cluster** | **Nb Vx Cluster** | **%Label** | **Nb Vx Label** |
| --- | --- | --- | --- | --- |
| Temporal_Mid_L | 86.15 | 65 | 1.13 | 4942 |
| Temporal_Inf_L | 13.85 | 65 | 0.28 | 3200 |
| Parietal_Inf_L | 100. 00 | 22 | 0.90 | 2447 |

**Supplementary Table 6. Volume summary of voxel-wise imagine analysis (^18^F-florzolotau PET; EOAD >LOAD)**

| **Label** | **%Cluster** | **Nb Vx Cluster** | **%Label** | **Nb Vx Label** |
| --- | --- | --- | --- | --- |
| Frontal_Sup_2_L | 100. 00 | 43 | 0. 88 | 4870 |
| Precuneus_1_L | 37.20 | 1817 | 19.16 | 3528 |
| Cingulate_Mid_R | 15.74 | 1817 | 12. 98 | 2203 |
| Cingulate_Mid_ L | 15. 24 | 1817 | 14.27 | 1941 |
| Precuneus_R | 13.81 | 1817 | 7. 69 | 3265 |
| Cingulate_Post_L | 10. 68 | 1817 | 41.90 | 463 |
| Cingulate_Post_R | 4.29 | 1817 | 23.28 | 335 |
| Cuneus_ L | 1. 54 | 1817 | 1. 84 | 1521 |
| Parietal_Sup_L | 0.28 | 1817 | 0.24 | 2065 |
| Occipital_Sup_L | 0.11 | 1817 | 0.15 | 1366 |
| Angular_L | 53. 44 | 262 | 11. 94 | 1173 |
| Occipital_Mid_L | 29.01 | 262 | 2.33 | 3265 |
| Parietal_Inf_L | 16. 41 | 262 | 1.76 | 2447 |
| Temporal_Mid_L | 1. 15 | 262 | 0.06 | 4942 |
| Frontal_Mid_2_R | 96.30 | 27 | 0.53 | 4860 |
| Frontal_Mid_2_L | 81. 82 | 11 | 0.20 | 4507 |
| Frontal_Inf_Oper_L | 9.09 | 11 | 0.10 | 1038 |
| Precentral_L | 9.09 | 11 | 0.03 | 3526 |
| SupraMarginal R | 39. 29 | 28 | 0.56 | 1974 |
| Parietal_Inf_R | 35. 71 | 28 | 0.74 | 1345 |
| Angular_R | 25.00 | 28 | 0.40 | 1752 |

**Supplementary Table 7. Volume summary of voxel-wise imagine analysis (^18^F-FDG PET; EOAD >LOAD)**

| **Label** | **%Cluster** | **Nb Vx Cluster** | **%Label** | **Nb Vx Label** |
| --- | --- | --- | --- | --- |
| Fusiform_R | 63.18 | 201 | 5. 04 | 2518 |
| Para_Hippocampal_R | 25.37 | 201 | 4. 51 | 1132 |
| Temporal_Inf_R | 10.45 | 201 | 0.59 | 3557 |
| Insula_R | 48.76 | 121 | 3. 33 | 1770 |
| Putamen_R | 8.26 | 121 | 0. 94 | 1062 |
| Cerebelum_6_L | 71. 83 | 142 | 6. 02 | 1694 |
| Cerebelum_4_5_ L | 11.97 | 142 | 1. 51 | 1125 |
| Cerebelum_Crus_1_L | 9.15 | 142 | 0.50 | 2603 |
| Fusiform L | 4.23 | 142 | 0.26 | 2309 |
| Para_Hippocampal_L | 2. 82 | 142 | 0. 41 | 978 |
| Rectus_R | 24.90 | 506 | 16. 91 | 745 |
| OFC_med_R | 22.92 | 506 | 18. 68 | 621 |
| Insula_R | 16. 80 | 506 | 4.80 | 1770 |
| OFCpost_R | 12. 45 | 506 | 11.23 | 561 |
| Frontal_Inf_Orb_2_R | 5.93 | 506 | 3.43 | 874 |
| Frontal_Inf_TriR | 5. 73 | 506 | 1. 35 | 2151 |
| Rectus_L | 5.53 | 506 | 3.29 | 852 |
| OFCant_R | 3. 56 | 506 | 2.78 | 648 |
| Supp_Motor_Area_L | 26. 00 | 250 | 3. 03 | 2147 |
| Cingulate_Mid_L | 23. 60 | 250 | 3. 04 | 1941 |
| Supp_Motor_Area_R | 18.80 | 250 | 1. 98 | 2371 |
| Frontal_Sup_Medial_L | 14. 80 | 250 | 1. 24 | 2992 |
| ACC_sup_L | 10.00 | 250 | 4.13 | 605 |
| ACC_pre_L | 6. 80 | 250 | 2.71 | 627 |
| Insula_L | 66.67 | 51 | 1.83 | 1858 |
| Frontal_Inf_Orb_2_L | 17. 65 | 51 | 1. 11 | 814 |
| OFCpost L | 15. 69 | 51 | 1. 41 | 567 |
| ACC_pre_R | 50.57 | 261 | 20.37 | 648 |
| Cingulate_Mid_R | 26.44 | 261 | 3.13 | 2203 |
| Frontal_Sup_Medial_R | 11. 49 | 261 | 1.41 | 2134 |
| ACC_sup_R | 9. 58 | 261 | 4. 69 | 533 |
| ACC_sup_L | 1.53 | 261 | 0. 66 | 605 |
| Frontal_Med_Orb_R | 0.38 | 261 | 0.12 | 856 |
| Cerebelum_6_R | 72. 73 | 11 | 0. 45 | 1795 |
| Cerebelum_Crus_1_R | 27.27 | 11 | 0. 11 | 2648 |

**Supplementary Figure 1. Group comparison of amyloid and tau PET SUVR between EOAD and LOAD group**

**
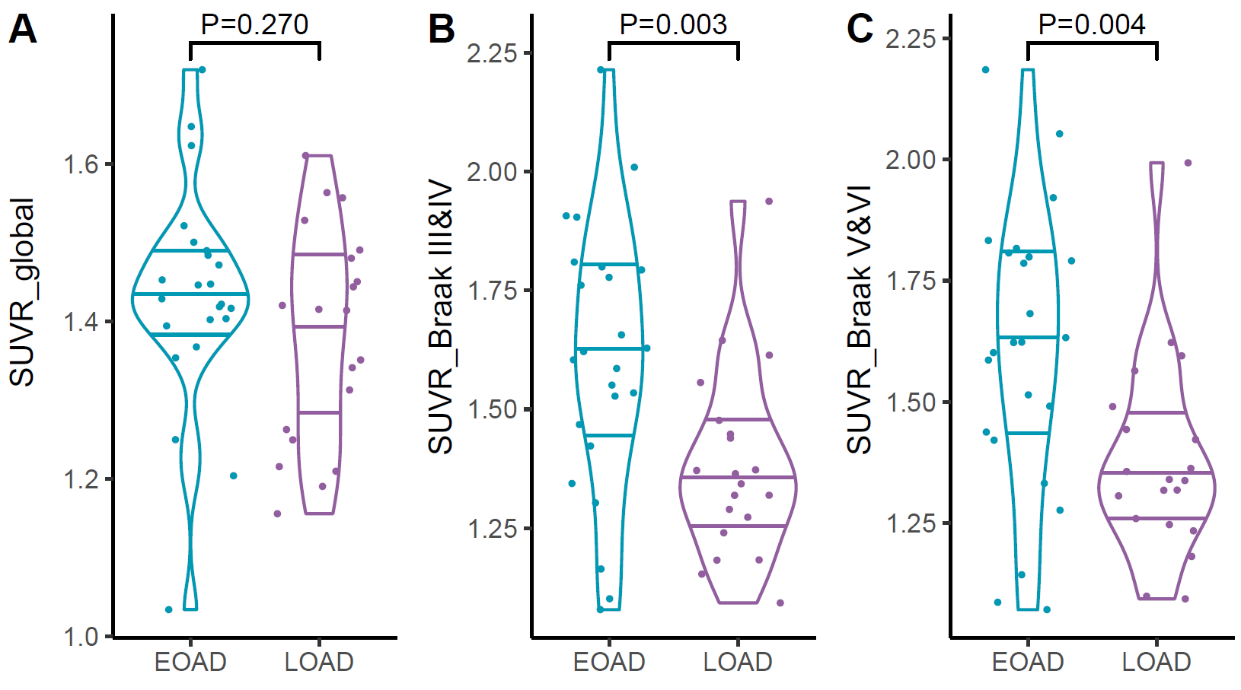
**

1. Global ^18^F-florbetapir PET SUVR in the EOAD and LOAD group; B. ^18^F- florzolotau PET SUVR in the Braak Ⅲ&Ⅳ regions; C. ^18^F- florzolotau PET SUVR in the Braak Ⅴ&Ⅵ regions.
